# Graft survival and mortality outcomes after kidney transplant in patients with lupus nephritis: a systematic review and meta-analysis

**DOI:** 10.1080/0886022X.2023.2296000

**Published:** 2024-01-04

**Authors:** Weizhong Jiang, Yunfen Xu, Qichun Yin

**Affiliations:** Department of Nephrology, Huzhou Central Hospital, Affiliated Central Hospital HuZhou University, Huzhou City, Zhejiang Province, China

**Keywords:** Lupus nephritis, SLE, renal transplant, living donor, deceased donor, meta-analysis

## Abstract

To explore the effect of lupus nephritis (LN) on graft survival in renal transplant patients. Literature search was conducted in PubMed, EMBASE and Scopus database for randomized controlled trials (RCTs), cohort, and case-control studies. The target population of interest was adult patients (aged >18 years) with end-stage renal disease (ESRD) and no history of previous renal transplants. Primary outcomes of interest were graft survival and patient survival. Pooled effect estimates were calculated using random-effects models and reported as hazard ratio (HR) with 95% confidence intervals (CI). A total of 15 studies were included. Compared to patients with ESRD due to other causes, patients with LN undergoing kidney transplant had lower patient survival rate (HR 1.15, 95% CI: 1.01, 1.31; *N* = 15, I^2^=34.3%) and worse graft survival (HR 1.06, 95% CI: 1.01, 1.11; *N* = 16, I^2^=0.0%), especially when studies with deceased donor were pooled together. Studies with a larger sample size (>200) showed that LN was strongly associated with lower graft and patient survival rates. Elevated risk of mortality in LN patients was detected in case-control studies, but not RCTs. On the other hand, RCTs, but not case-control studies, showed an increased risk of poor graft survival in LN patients. The findings suggest that the presence of LN might have a negative impact on both the graft survival and the overall patient survival of post-transplant ESRD patients. Further studies that account for factors such as study methodology, donor characteristics, and sample size are needed to reach definitive conclusions. Renal transplant patients with LN should undergo regular follow-up examinations.

## Introduction

Systemic lupus erythematosus (SLE) is a complex autoimmune disease that can affect various systems and organs within the body, including the kidneys [1,12]. Kidney involvement, known as lupus nephritis (LN), is a significant complication of end-stage renal disease SLE. It’s estimated that about 40-50% of patients with SLE will develop LN [4,14] which significantly contributes to the overall poor prognosis of SLE patients. Lupus nephritis presents a substantial challenge to clinicians due to its unpredictable disease course. Up to 10-30% of patients may progress to end-stage renal disease (ESRD) within 15 years of their LN diagnosis [7,35].

Kidney transplantation has emerged as a viable treatment option for patients with LN who progress to ESRD [17]. However, the intricate interplay between lupus-related immune dysregulation, post-transplant immunosuppression, and the risk of graft survival and patient mortality requires a comprehensive evaluation to guide clinical decision-making.

Despite advances in immunosuppressive therapies and transplant techniques, the outcomes of kidney transplantation in patients with LN remain unclear. While some reports indicate comparable graft survival rates and patient survival outcomes in this population when compared to patients with ESRD of other etiology, others show evidence of the increased risks of acute rejection, graft loss, and mortality in recipients with a history of lupus nephritis [6,9,28,30,38,40]. A review and meta-analysis of Kim et al. that included 17 studies of graft survival rates in renal transplant LN patients [16], reported similar 1- and 5-year graft survival rates in LN and control recipients. However, several new studies have been published since then.

This meta-analysis aims to provide the most updated and contemporary evidence on the association of LN with graft survival in ESRD patients undergoing renal transplant.

## Methods

### Primary objective

The primary objective of the study was to systematically analyze available data from relevant studies to assess the connection between lupus nephritis (LN) and the outcomes of patient and graft survival. The reference group comprised kidney transplant patients with ESRD of etiology not related to LN.

### Search for potential literature

Systematic literature search of databases such as PubMed, Embase, and Scopus was done for studies published until July 31, 2023. The protocol of our study was registered at PROSPERO, CRD42023454957. The search criteria incorporated relevant keywords, synonyms, and Medical Subject Headings (MeSH) terms. Our search strategy consisted of a combination of keywords: (lupus nephritis OR SLE OR autoimmune OR Lupus Erythematosus) AND (renal transplant OR kidney transplant) AND (clinical outcomes OR patient survival OR graft survival OR overall survival). The search terms used in the three databases have been presented as Supplementary Table 1. We applied search filters or limits related to publication date (year 2000 and onwards), article type (observational studies and controlled clinical trials), species (humans) and age (adult 19+ years). We performed manual searches of the reference lists and relevant review articles to identify any potentially relevant studies.

### Inclusion and exclusion criteria

Original research studies of various study designs were included, such as prospective and retrospective cohort studies, case-control studies, and randomized controlled trials (RCTs). The target population of interest was adult patients (aged >18 years) who have undergone kidney transplantation for ESRD due to lupus nephritis (LN), while the reference group consisted of adult patients who received kidney transplants for ESRD of other etiology. The primary outcome of interest was graft survival and patient survival.

Inclusion criteria were as follows: 1) Studies that had reported the outcome of interest at least 12 months post-transplant; 2) Studies published until July 31, 2023; 3) Studies that were published after the year 2000, to account for recent advancements in transplantation techniques, immunosuppression, and post-transplant care. In cases of studies with different timepoints of follow up, only data reported at the latest follow up were used.

Exclusion criteria were as follows: 1) Patients with previous renal transplants; 2) Studies involving animal experiments, conference abstracts, letters, case-reports, editorials, and reviews; 3) Studies exclusively involving pediatric and adolescent patients (aged under 18 years), those with incomplete or inadequate outcome data, interventions other than kidney transplantation, and studies unrelated to the research question.

In cases of multiple publications from the same study, only the most comprehensive or recent publication was included to prevent data duplication.

### Study selection, data extraction and quality assessment

After applying our search strategy across the three databases and generating the initial pool of studies, duplicate studies were removed. Two authors independently screened the remaining studies. In the initial screening phase, titles and abstracts were evaluated for relevance. In the subsequent phase, an in-depth evaluation of the full texts of the selected studies were done. Any discrepancies were resolved through thorough discussion, and when necessary, a third author’s perspective was sought. This study adhered to the PRISMA guidelines [27]. Additionally, the study protocol was registered in PROSPERO with a unique registration number.

Data extraction was carried out using a standardized data extraction form that included key study characteristics, such as a year of publication, place of conduct, patient demographics, sample size, duration of follow up, type of transplantation (living or deceased donor) and findings on outcomes of interest. Data extraction process was carried out independently by two reviewers, with any disparities resolved through thorough discussion and consensus. In cases where discrepancies remained unresolved, a third reviewer was consulted to make the final decision. We used the Newcastle-Ottawa Scale (NOS) for quality assessment of the included studies [36]. It is commonly used in systematic reviews and meta-analyses to assess the quality of non-randomized studies, such as cohort and case-control studies. It aids the researchers and reviewers in evaluating the risk of bias and to determine the overall methodological quality. The NOS typically includes several criteria that address various aspects of study design and execution, such as participant selection, comparability, and outcome assessment. It assigns a numerical score to each study and a higher NOS score generally indicates a study with lower risk of bias and better methodological quality. Additionally, we also used the ROBINS-I tool (“Risk Of Bias In Non-randomised Studies – of Interventions”) for assessing quality of the included studies [34]. We used *“robvis”* for visualizing risk-of-bias assessments and create risk-of-bias plots [20].

### Statistical analysis

Pooled effect sizes were reported as Hazards ratio (HR) along with 95% confidence interval (CI). We decided to use the random effects model, using the Restricted Maximum Likelihood Method (REML). The included studies differed in their methodology (for instance, study design, sample size, duration of follow up, participant characteristics, donor characteristics etc). These differences were bound to create substantial heterogeneity and therefore, random effects model was considered apt for the analysis and this was used to generate forest plots. Publication bias was assessed using Egger’s test and through visual inspection of funnel plot [11]. Subgroup analysis was done based on the type of donor (living and deceased), sample size (≤200 and >200) and type of study design. For all analysis, a p-value of less than 0.05 was considered to indicate a statistical significance.

## Results

**Figure 1.**
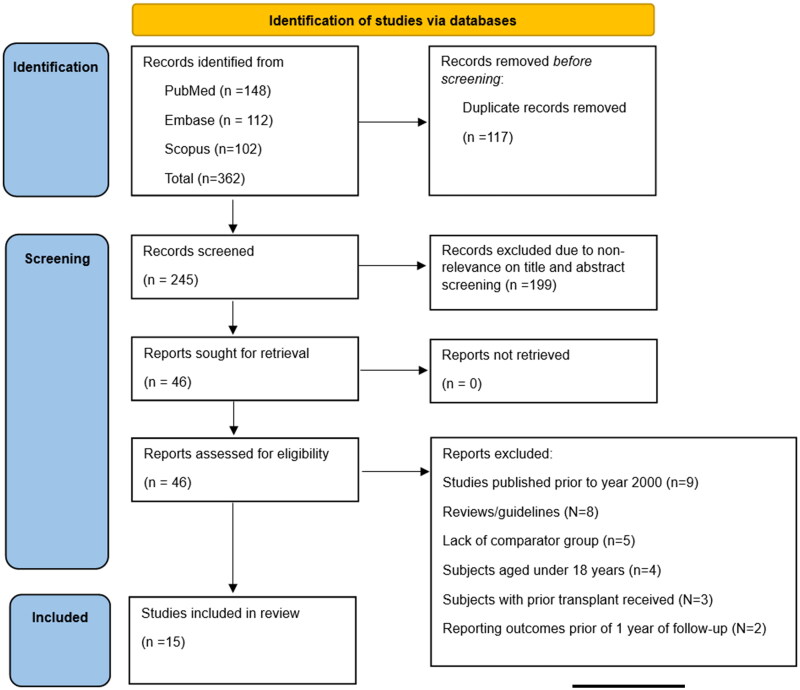
Selection process flowchart.

Systematic search across the databases identified 362 studies. After eliminating 117 duplicate studies, 245 papers (Figure 1) underwent an initial titles and abstracts assessment, and additional 199 were excluded. Full texts of the remaining 46 studies were screened. Finally, 15 studies [5,6,8,9,15,16,19,21,23–25,28,30,38,40] were included in the meta-analysis. The studies that were excluded at the time of full-text review have been presented in Supplementary table 2.

In the initial screening exercise with 80 records, the inter-rater reliability was 74.1%. This was mainly due to lack of clarity about the exclusion criteria among the two authors. Further to this, a detailed discussion was scheduled with the authors involved with the screening and selection of studies. The discussion was led by the senior experienced author and an explicitly written inclusion and exclusion criteria was provided to both the authors. Finally, out of the 245 unique studies screened with inclusion of 15 studies, the inter-rater reliability showed an agreement of 95.2%.

For the extraction of data from the initial 8 studies, the percent agreement was 83.0%. The researchers involved in data extraction had different interpretations of the data presented in the original studies. There was also discrepancy and variability in the assessment of study quality and risk of bias. As a response to this issue, a comprehensive discussion was arranged involving the authors responsible for data extraction and quality assessment. This discussion was chaired by the senior, more experienced author. Toward the end of the data extraction exercise, complete agreement was achieved.

Out of the 15 studies, 7 had a retrospective cohort design and the remaining 8 were case-control studies (Table 1). Two studies each were conducted in Mexico, South America, and United States. One study each was conducted in Spain, South Korea, Taiwan, Greece, Italy, Norway, and Netherlands. There were two multicentric studies. Majority of the studies (*n* = 12; 80%) had a higher proportion of female subjects. A total of 10 studies involved use of cadaveric/deceased donor. Living donor was used in 4 studies. One study utilized both living and deceased donor and had presented data on renal transplant outcomes separately for these two types of donors [5]. The timing of outcome assessment post-transplant ranged from 36 months to >10 years (Table 1). The primary causes of renal transplant in patients with non-lupus related ESRD were idiopathic glomerulonephritis, diabetic nephropathy, pyelonephritis, renal hypoplasia, hypertensive nephropathy, polycystic kidney disease, Ig A nephropathy, focal segmental glomerulosclerosis, and hemolytic uremic syndrome (Table 1). The total number of patients in all 15 studies was 1,74,985 (6456 with LN and 1,68,529 with non-LN related ESRD). NOS quality assessment score ranged from 7 to 9 (out of the maximum attainable score of 9), with a mean score of 7.6. The findings of the assessment based on ROBINS-I tool have been presented as Supplementary figure 1. The assessment indicated that the included studies were of acceptable quality with no critical risk of bias.

**Table 1.** Included studies.

Author (year of publication)	Study design	Country	Subject characteristics	Sample size	Newcastle Ottawa quality score
[9]	Retrospective cohort (registry data)	Multicentric (Europe)	Subjects (with or without SLE) matched for baseline characteristics; mean age at transplant ∼40 years; females (82%); majority with deceased donor (∼70%) Follow up of around 10 years No significant difference in immunosuppressive regimens between both groups Primary etiology in those without lupus nephritis (LN): glomerulonephritis (19.5%), Diabetic nephropathy (20%) and pyelonephritis (10.2%)	SLE: 999 No SLE: 4995	9
[16]	Retrospective cohort	South Korea	Mean age at transplant ∼32 years; females (75%); cadaver/deceased donor in >50%; Median post-transplant follow up of around 9.5 years Data not provided on primary etiology in those without lupus nephritis (LN).	SLE: 20 No SLE: 50	7
[15]	Retrospective cohort	Mexico	Median age at transplant ∼27 years; females (75%); majority with living donor relative (∼70%) Median post-transplant follow up of around 36 months No significant difference in immunosuppressive regimens between both groups Primary etiology in those without lupus nephritis (LN): Idiopathic glomerulonephritis (78%) and renal hypoplasia (6%)	SLE: 25 No SLE: 50	7
[28]	Case-control	Spain	Median age at transplant ∼40 years; females (35%); cadaver/deceased donor in all; Median post-transplant follow up of around 105 months Primary etiology in those without lupus nephritis (LN): primary glomerulonephritis.	SLE: 43 No SLE: 367	7
[30]	Case-control	Mexico	Median age at transplant of around 32 years; female (83%) Majority with living donor (66%); Follow up period of around 11 years. Primary etiology in those without lupus nephritis (LN): chronic glomerulonephritis, congenital urinary abnormality, polycystic kidney disease and diabetic nephropathy.	SLE: 74 No SLE: 148	8
[23]	Case-control	South America	Mean age at transplant of around 34 years; female (85%); Majority with deceased donor (>70%); Median follow up period of 90 months Data not provided on primary etiology in those without lupus nephritis (LN).	SLE: 65 No SLE: 65	7
[40]	Retrospective cohort (registry data)	Multicentric (Australia and New Zealand)	Mean age at transplant of around 40 years; female (80% in SLE and 37% in non-SLE group); Majority with deceased donor (>50%); Median follow up period of 3.8 years No significant difference in immunosuppressive regimens between both groups Data not provided on primary etiology in those without lupus nephritis (LN).	SLE: 176 No SLE: 8541	8
[24]	Retrospective cohort	South America	Mean age at transplant of around 33 years in those with SLE and 50 years in non-SLE group; female (88% in SLE and 30% in non-SLE); Majority with cadaveric donor (99%); Follow up period of around 5 years. Primary etiology in those without lupus nephritis (LN): polycystic kidney disease and diabetic nephropathy.	SLE: 27 No SLE: 109	8
[25]	Case-control	Norway	Mean age at transplant of around 37 years; female (80%); Majority with living donor (>50%); Follow up period of >10 years No significant difference in immunosuppressive regimens between both groups Primary etiology in those without lupus nephritis (LN): membranoproliferative glomerulonephritis; IgA nephropathy and focal segmental glomerulosclerosis	SLE: 77 No SLE: 154	8
[38]	Case-control	Taiwan	Mean age at transplant of around 34 years; female (>70%); Majority deceased donor (>90%); Mean follow up period of 107 months No significant difference in immunosuppressive regimens between both groups Data not provided on primary etiology in those without lupus nephritis (LN).	SLE: 23 No SLE: 94	8
[19]	Case-control	Greece	Mean age at transplant of around 35 years; female (89%); Majority living donor (54%); Mean follow up period of 80 months Primary etiology in those without lupus nephritis (LN): chronic glomerulonephritis (35%); chronic pyelonephritis (21%); polycystic kidney disease (14%) and reflux nephropathy (14%).	SLE: 26 No SLE: 26	7
[6]	Retrospective cohort	United States	Mean age of around 43 years; female (40%); Majority deceased donor (75%); Mean follow up period of 4.7 years No significant difference in immunosuppressive regimens between both groups Primary etiology in those without lupus nephritis (LN): glomerulonephritis (26%); diabetic nephropathy (25%) and hypertensive nephropathy (17%).	SLE: 2886 No SLE: 89,958	8
[5]	Retrospective cohort	United States	Majority of the subjects were aged 22 to 60 years (∼80%); females (∼40%); Both cadaver/deceased donor and living donor; Follow up of 5 years No significant difference in immunosuppressive regimens between both groups Data not provided on primary etiology in those without lupus nephritis (LN).	*Cadaveric donor*SLE: 1170 No SLE: 42,651 *Living donor*SLE: 789 No SLE: 21,228	8
[21]	Case-control	Italy	Mean age at transplant of around 36 years; female (80%) Majority with cadaveric donor (80%); Follow up period of around 90 months. Primary etiology in those without lupus nephritis (LN): membranoproliferative glomerulonephritis; IgA nephropathy, hypertensive nephropathy, polycystic kidney disease and focal segmental glomerulosclerosis	SLE: 33 No SLE: 70	7
[8]	Case-control	Netherlands	Mean age at transplant of around 35 years; female (90%) Majority with cadaveric donor (83%); Follow up period of around 80 months. Primary etiology in those without lupus nephritis (LN): chronic pyelonephritis, hemolytic uremic syndrome, membranoproliferative glomerulonephritis; IgA nephropathy, interstitial nephritis, nephropathy and polycystic kidney disease	SLE: 23 No SLE: 23	7

### Overall patient survival

**Figure 2.**
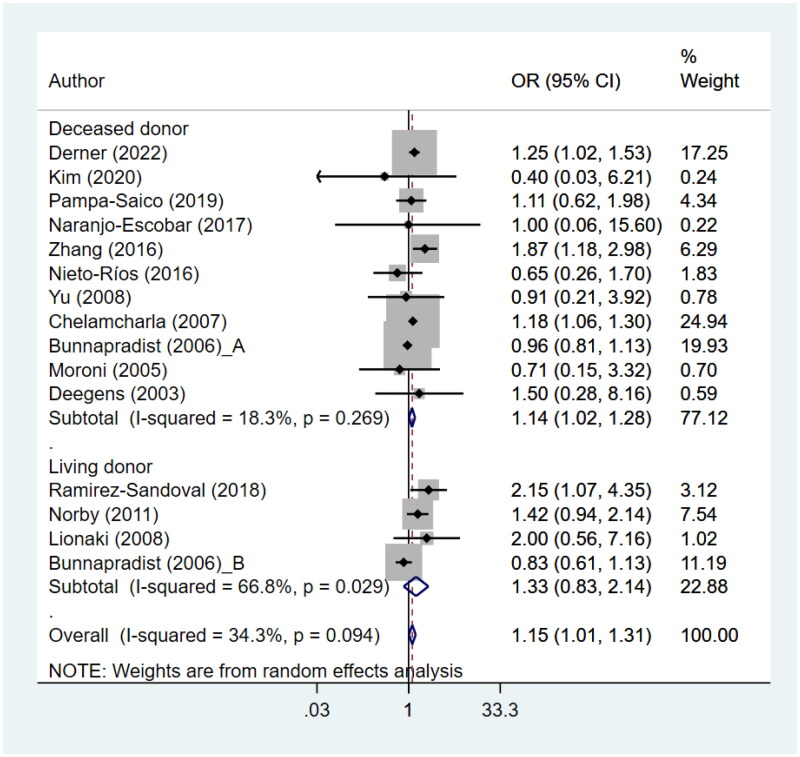
Patient survival.

ESRD patients with LN undergoing kidney transplant had poor patient survival (HR 1.15, 95% CI: 1.01, 1.31; *N* = 15, I^2^=34.3%) compared to patients in the reference group (Figure 2). There was no evidence of publication bias, both of egger test (*p* = 0.89) and on visual inspection of funnel plot (Supplementary figure 2). Categorizing the data according to the type of donor indicated that the risk of poor patient survival was statistically significant when studies involving cadaveric/deceased donors were combined (HR 1.14, 95% CI: 1.02, 1.28; *N* = 11, I^2^=18.3%) (Figure 2). However, the risk was not significant when studies involving living donors were pooled together (HR 1.33, 95% CI: 0.83, 2.14; *N* = 4, I^2^=66.8%). The common reported reasons for patient mortality were adverse cardiovascular events, septicaemia/infection, septic shock, and malignancy.

A subgroup analysis based on the size of the sample showed that pooling together studies with a larger sample size (>200) resulted in a significant association between presence of LN and poor patient survival (HR 1.18, 95% CI: 1.01, 1.37; *N* = 8, I^2^=61.1%) (Table 2, Supplementary figure 3). However, studies with relatively smaller sample sizes (≤200) did not demonstrate a significant association (HR 0.93, 95% CI: 0.53, 1.62; *N* = 7, I^2^=0.0%). Studies with a retrospective cohort design did not display a significantly elevated risk of poor survival with presence of LN (HR 1.10, 95% CI: 0.93, 1.30; *N* = 7, I^2^=61.6%) (Table 2, Supplementary figure 4). On the other hand, studies following a case-control design, when combined, indicated an increased risk of poor survival in LN patients (HR 1.40, 95% CI: 1.06, 1.85; *N* = 8, I^2^=0.0%) (Table 2, Supplementary figure 4).

**Table 2.** Subgroup analysis.

	Overall patient survival	Graft survival
	HR (95% Confidence interval) (N; I^2^)	
Sample size		
≤200	0.93 (0.53, 1.62) (N = 7; I^2^=0.0%)	1.28 (0.96, 1.72) (N = 8; I^2^=0.0%)
>200	1.18 (1.01, 1.37) (N = 8; I^2^=61.1%) *	1.05 (1.01, 1.11) (N = 8; I^2^=0.0%) *
Study design		
Retrospective cohort	1.10 (0.93, 1.30) (N = 7; I^2^=61.6%)	1.06 (1.01, 1.12) (N = 8; I^2^=0.0%) *
Case-control	1.40 (1.06, 1.85) (N = 8; I^2^=0.0%) *	1.01 (0.83, 1.24) (N = 8; I^2^=0.0%)

*Statistically significant at *p* < 0.05.

### Graft survival

**Figure 3.**
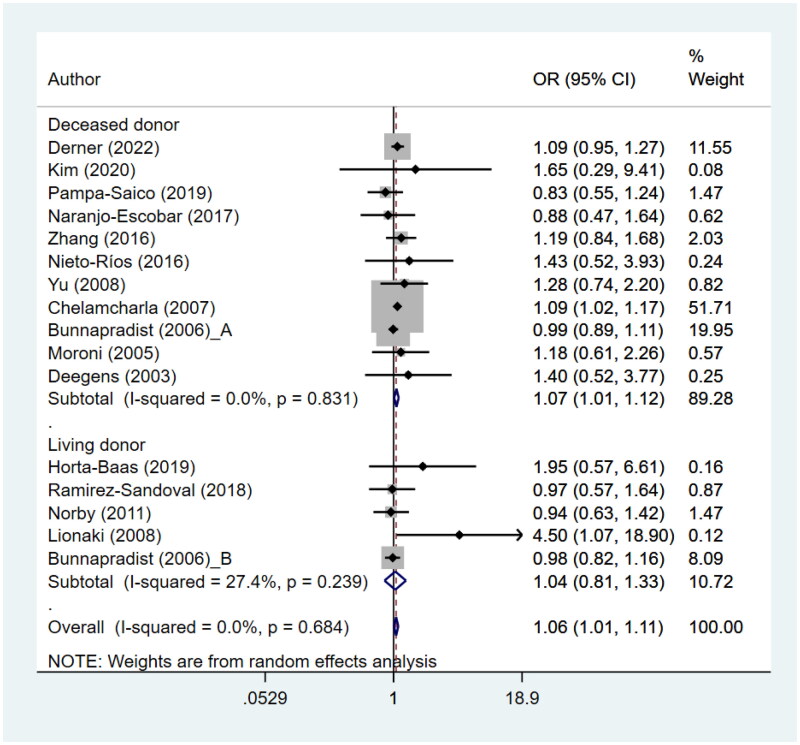
Graft survival.

Patients with LN undergoing kidney transplant had poor graft survival (HR 1.06, 95% CI: 1.01, 1.11; *N* = 16, I^2^=0.0%), compared to patients with ESRD due to other causes (Figure 3). There was no evidence of publication bias, both of egger test (*p* = 0.32) and on visual inspection of funnel plot (Supplementary figure 5). The risk of poor graft survival was statistically significant when studies involving cadaveric/deceased donors were combined (HR 1.07, 95% CI: 1.01, 1.12; *N* = 11, I^2^=0.0%) but not when studies involving living donors were pooled together (HR 1.04, 95% CI: 0.81, 1.33; *N* = 5, I^2^=27.4%) (Figure 3). The reported reasons for graft failure were acute rejection, infection by BK virus, chronic allograft nephropathy, acute graft arterial occlusion and renal vein thrombosis.

Subgroup analysis based on the size of the sample showed that studies with a larger sample size (>200) had a significant association between presence of LN and poor graft survival (HR 1.05, 95% CI: 1.01, 1.11; *N* = 8, I^2^=0.0%) (Table 2, Supplementary figure 6). However, studies with relatively smaller sample sizes (≤200) did not demonstrate a significant association (HR 1.28, 95% CI: 0.96, 1.72; *N* = 8, I^2^=0.0%). Pooling of findings from studies with a retrospective cohort design showed an association of the increased risk of poor graft survival with the presence of LN (HR 1.06, 95% CI: 1.01, 1.12; *N* = 8, I^2^=0.0%). However, this association was not significant when studies with a case-control design were pooled (HR 1.01, 95% CI: 0.83, 1.24; *N* = 8, I^2^=0.0%) (Table 2, Supplementary figure 7).

## Discussion

The results of this meta-analysis showed an increased risk of patient mortality and graft failure in kidney transplant recipients with LN, compared to patients with ESRD of another etiology. This increased risk was limited to those receiving cadaveric/deceased donor. The findings have immense clinical significance. Clinicians and patients need to be aware of this increased risk when considering kidney transplantation for individuals with LN. It underscores the importance of discussing the benefits and risks associated with the choice of donor source. Living donor transplantation may offer a better outcome, and healthcare providers should consider this option when possible. Patients with LN should receive comprehensive counseling about the potential risks associated with different donor sources. These results may influence healthcare resource allocation, as they could lead to a reconsideration of transplant organ allocation policies and strategies to optimize graft and patient survival in LN patients. Physicians and healthcare teams may need to intensify post-transplant care and surveillance for kidney transplant recipients with LN, particularly those who received cadaveric donor kidneys, to detect and manage complications early.

Other studies suggested that the systemic nature of LN, involving not only the kidneys but also other organ systems, may contribute to higher post-transplant mortality rates due to potential complications and challenges in managing comorbidities [22,31]. Our analysis showed an increased risk of graft failure in patients with LN. This finding is in agreement with previous studies and further underscores the complex immune milieu associated with LN, which might impact graft viability [2,26,29]. Autoimmune processes and ongoing immune activity in LN patients could potentially contribute to chronic graft inflammation [10] and dysfunction [2,29]. Our results suggest that optimizing immunosuppressive regimens [3,39], managing potential autoimmune flare-ups, and closely monitoring graft function is crucial in improving graft survival in this specific patient subgroup. One key observation to note is that while the current study shows an association between LN and adverse outcomes, this cannot be considered as causative and future mechanistic studies are needed to confirm causality.

The differences in the observed outcomes between studies involving deceased donors and those involving living donors in the context of patient mortality and graft rejection rates raises several intriguing questions. While the exact reasons for this discrepancy need further investigation, several potential factors could contribute to this phenomenon. Deceased donor kidneys often come from individuals with traumatic injuries or certain medical conditions that could impact the quality of the organ [33]. This might lead to a higher likelihood of immune challenges during the initial phase of graft adaptation in recipients. The immune response triggered by a deceased donor graft might be more robust due to increased antigenicity, which could contribute to a higher risk of graft rejection and associated patient mortality [37]. On the other hand, living donor kidneys are typically procured from healthy individuals, resulting in a potentially more favorable immunological milieu that promotes graft acceptance and better outcomes. The quality of the donor organ can play a significant role in transplant outcomes. Deceased donor kidneys might have a higher risk of ischemic injury, longer preservation times, and more exposure to potential damaging factors prior to transplantation that may impact their quality [41]. These factors could negatively impact the initial graft function and contribute to subsequent complications. Additionally, living donor kidney transplantation allows for a higher degree of compatibility between the donor and recipient due to controlled donor selection and better matching [13]. This reduces the risk of immune-mediated complications and increases the odds of successful graft integration. In contrast, in case of using a deceased donor kidney, there is always a risk of suboptimal matching due to factors like time constraints, organ availability, and the need to allocate organs to a broader recipient pool [18,32]. Such mismatches could contribute to a higher risk of graft rejection and potentially poorer outcomes. To fully understand the observed disparity between studies involving deceased and living donors, future research should delve into the specific factors related to each donor type that influence transplant outcomes. Analyzing donor characteristics, immune responses, graft quality, and long-term survival patterns in a more granular manner could provide valuable insights.

We found that only case-control studies showed a significantly elevated risk of poor survival with the presence of LN. On the other hand, only studies with a retrospective cohort design showed an association of the increased risk of poor graft survival with the presence of LN. These variations in associations observed when comparing different study designs i.e. retrospective cohort and case-control studies, can often be attributed to the inherent strengths and limitations of each design, such as differences in the way data is collected, the level of control over confounding variables, and the temporal sequence of exposure and outcome assessment. When studying associations between LN and kidney transplantation outcomes in retrospective cohort studies, variations might arise due to the challenges of accurately capturing the timing of exposure (presence of LN) and the temporal sequence of transplant outcomes. Additionally, unmeasured confounding variables can affect the observed associations. For example, patient characteristics such as disease severity, comorbidities, and overall health might not be adequately accounted for, leading to potential confounding and variations in associations. Case-control studies, on the other hand, are prone to selection bias, recall bias, and issues related to the representativeness of the control group.

The results of our meta-analysis have important clinical implications for the management of kidney transplant recipients with LN. Our results suggest that these patients may require tailored strategies to mitigate the increased risk of patient mortality and graft failure. Multidisciplinary care involving rheumatologists, nephrologists, and transplant specialists is essential to address both systemic autoimmune manifestations and transplant-related factors. Further research is needed to understand the underlying mechanisms that contribute to the observed differences in outcomes. Longitudinal studies assessing factors such as immunosuppression protocols, post-transplant immune reactivity, and the impact of autoimmune activity on graft function may provide a more nuanced understanding of the challenges faced by LN recipients. Additionally, the potential role of genetic factors and the impact of different lupus nephritis subtypes on transplant outcomes need to be explored.

There are some limitations of our review. First, the studies included in the meta-analysis had different patient populations in terms of disease severity, comorbidities, and demographics. LN itself is a heterogeneous condition with various manifestations and outcomes. The variability in patient characteristics across studies may have introduced clinical heterogeneity. Clinical and methodological heterogeneity across studies in terms of participant characteristics, interventions, follow-up periods, and outcome definitions could have affected the reliability of the pooled results. Another limitation of our study is that we did not search for grey literature or trial registers. Doing so would have minimized the possibility of publication bias. Retrospective studies might be subject to recall bias, selection bias, and confounding, while case-control studies could have been influenced by issues like control selection and temporal sequencing. Additionally, some included studies had small sample sizes, and some did not uniformly control for important confounding variables, such as immunosuppressive regimens, comorbidities, disease severity and socioeconomic factors. Uncontrolled or inadequately controlled confounding could have impacted the observed associations between LN and graft survival/mortality outcomes. Future studies should adopt a proper statistical analysis plan and adjust for important confounders. The timing of graft survival and mortality outcome assessment varied across studies. Some studies had longer follow-ups, allowing them to capture long-term outcomes more accurately. The differences in follow-up durations could also have influenced the observed effect sizes. In our study, the majority of studies were from specific geographical regions, limiting global generalization. There is a need for multi-centric trials that could make the findings applicable to a large geographical setting. Another limitation was that the donor characteristics were not analyzed, mostly because of limited information on this aspect in the included studies. These limitations call for future multi-centric research to strive for more homogenous cohorts to improve result pooling and provide reliable evidence. Further, as many of the available studies failed to adequately control for potential confounders, addressing this through proper study design and analysis is essential in future research.

## Conclusion

Our findings highlight the increased risks of patient mortality and graft failure in kidney transplant recipients with LN compared to those with ESRD from other causes. However, given the limitations, definitive conclusions cannot be drawn yet. More robust studies, considering factors such as study methodology, donor characteristics and sample size, are needed to confirm our conclusions. The definitions and methods used to assess outcomes should be standardized across studies in order to make the findings harmonized and relevant across larger geographical context. Retrospective and case-control studies have inherent biases that need to be limited in future research. Also, future studies should be conducted with large sample sizes that provide adequate power and with sufficiently long follow up period to arrive at a reliable conclusion. Our findings further emphasize the need for personalized transplant care strategies that address the unique challenges posed by systemic autoimmune conditions like LN. A comprehensive approach, encompassing both immunosuppressive management and holistic patient care, is imperative to improve the long-term outcomes for this patient population.

## Supplementary Material

Supplemental Material

## References

[1] Ameer MA, Chaudhry H, Mushtaq J, et al. An overview of systemic lupus erythematosus (SLE) pathogenesis, classification, and management. Cureus. 2022;14(10):1. doi:10.7759/cureus.30330. PMC9662848 36407159

[2] Apostolidis SA, Lieberman LA, Kis-Toth K, et al. The dysregulation of cytokine networks in systemic lupus erythematosus. J Interferon Cytokine Res. 2011;31(10):769–10. doi:10.1089/jir.2011.0029. 21877904 PMC3189553

[3] Aringer M, Smolen JS. Efficacy and safety of TNF-blocker therapy in systemic lupus erythematosus. Expert Opin Drug Saf. 2008;7(4):411–419. doi:10.1517/14740338.7.4.411. 18613805

[4] Barber MRW, Drenkard C, Falasinnu T, et al. Global epidemiology of systemic lupus erythematosus. Nat Rev Rheumatol. 2021;17(9):515–532. doi:10.1038/s41584-021-00668-1. 34345022 PMC8982275

[5] Bunnapradist S, Chung P, Peng A, et al. Outcomes of renal transplantation for recipients with lupus nephritis: analysis of the organ procurement and transplantation network database. Transplantation. 2006;82(5):612–618. doi:10.1097/01.tp.0000235740.56573.c6. 16969282

[6] Chelamcharla M, Javaid B, Baird BC, et al. The outcome of renal transplantation among systemic lupus erythematosus patients. Nephrol Dial Transplant. 2007;22(12):3623–3630. doi:10.1093/ndt/gfm459. 17640941

[7] Croca SC, Rodrigues T, Isenberg DA. Assessment of a lupus nephritis cohort over a 30-year period. Rheumatology (Oxford). 2011;50(8):1424–1430. doi:10.1093/rheumatology/ker101. 21415024

[8] Deegens JKJ, Artz MA, Hoitsma AJ, et al. Outcome of renal transplantation in patients with systemic lupus erythematosus. Transpl Int. 2003;16(6):411–418. doi:10.1007/s00147-003-0563-9. 12819872

[9] Derner O, Kramer A, Hruskova Z, et al. Incidence of kidney replacement therapy and subsequent outcomes among patients with systemic lupus erythematosus: findings from the ERA registry. Am J Kidney Dis. 2022;79(5):635–645. doi:10.1053/j.ajkd.2021.09.016. 34752912

[10] Ding Y, Tan Y, Qu Z, et al. Renal microvascular lesions in lupus nephritis. Ren Fail. n.d;42(1):19–29. doi:10.1080/0886022X.2019.1702057. 31858861 PMC6968586

[11] Egger M, Davey Smith G, Schneider M, et al. Bias in meta-analysis detected by a simple, graphical test. BMJ. 1997;315(7109):629–634. doi:10.1136/bmj.315.7109.629. 9310563 PMC2127453

[12] Fava A, Petri M. Systemic lupus erythematosus: diagnosis and clinical management. J Autoimmun. 2019;96:1–13. doi:10.1016/j.jaut.2018.11.001. 30448290 PMC6310637

[13] Frutos MÁ, Crespo M, Valentín M, et al. Recommendations for living donor kidney transplantation. Nefrologia (Engl Ed). 2022;42(Suppl 2):5–132. doi:10.1016/j.nefroe.2022.07.001. 36503720

[14] Hoover PJ, Costenbader KH. Insights into the epidemiology and management of lupus nephritis from the US rheumatologist’s perspective. Kidney Int. 2016;90(3):487–492. doi:10.1016/j.kint.2016.03.042. 27344205 PMC5679458

[15] Horta-Baas G, Camargo-Coronel A, Miranda-Hernández DG, et al. Renal transplantation in systemic lupus erythematosus: comparison of graft survival with other causes of end-stage renal disease. Reumatol Clin (Engl Ed). 2019;15(3):140–145. doi:10.1016/j.reuma.2017.07.006. 28818581

[16] Kim JE, Kim YC, Min S-L, et al. Transplant outcomes in kidney recipients with lupus nephritis, and systematic review. Lupus. 2020;29(3):248–255. doi:10.1177/0961203320902524. 31996111

[17] Kostopoulou M, Fanouriakis A, Cheema K, et al. Management of lupus nephritis: a systematic literature review informing the 2019 update of the Joint EULAR and European Renal Association-European Dialysis and Transplant Association (EULAR/ERA-EDTA) recommendations. RMD Open. 2020;6(2):e001263. doi:10.1136/rmdopen-2020-001263. 32699043 PMC7425195

[18] Lee D, Kanellis J, Mulley WR. Allocation of deceased donor kidneys: a review of international practices. Nephrology (Carlton). 2019;24(6):591–598. doi:10.1111/nep.13548. 30536674

[19] Lionaki S, Kapitsinou PP, Iniotaki A, et al. Kidney transplantation in lupus patients: a case-control study from a single centre. Lupus. 2008;17(7):670–675. doi:10.1177/0961203308089430. 18625640

[20] McGuinness LA, Higgins JPT. Risk-of-bias VISualization (robvis): an R package and shiny web app for visualizing risk-of-bias assessments. Res Synth Methods. 2021;12(1):55–61. doi:10.1002/jrsm.1411. 32336025

[21] Moroni G, Tantardini F, Gallelli B, et al. The long-term prognosis of renal transplantation in patients with lupus nephritis. Am J Kidney Dis. 2005;45(5):903–911. doi:10.1053/j.ajkd.2005.01.038. 15861356

[22] Murimi-Worstell IB, Lin DH, Nab H, et al. Association between organ damage and mortality in systemic lupus erythematosus: a systematic review and meta-analysis. BMJ Open. 2020;10(5):e031850. doi:10.1136/bmjopen-2019-031850. PMC7247371 32444429

[23] Naranjo-Escobar J, Manzi E, Posada JG, et al. Kidney transplantation for end-stage renal disease in lupus nephritis, a very safe procedure: a single Latin American transplant center experience. Lupus. 2017;26(11):1157–1165. doi:10.1177/0961203317696591. 28420067

[24] Nieto-Ríos JF, Serna-Higuita LM, Builes-Rodriguez SA, et al. Clinical outcomes of kidney transplants on patients with end-stage renal disease secondary to lupus nephritis, polycystic kidney disease and diabetic nephropathy. Colombia Medica (Cali, Colombia). 2016;47(1):51–58. doi:10.25100/cm.v47i1.2085. 27226665 PMC4867517

[25] Norby GE, Leivestad T, Mjøen G, et al. Premature cardiovascular disease in patients with systemic lupus erythematosus influences survival after renal transplantation. Arthritis Rheum. 2011;63(3):733–737. doi:10.1002/art.30184. 21360503

[26] Ohmes J, Comdühr S, Akbarzadeh R, et al. Dysregulation and chronicity of pathogenic T cell responses in the pre-diseased stage of lupus. Front Immunol. 2022;13:1007078. doi:10.3389/fimmu.2022.1007078. 36389689 PMC9650673

[27] Page MJ, McKenzie JE, Bossuyt PM, et al. PRISMA. Transparent reporting of systematic reviews and meta-analyses. BMJ. 2021;372:n71. doi:10.1136/bmj.n71. 33782057 PMC8005924

[28] Pampa-Saico S, Marcén-Letosa R, Fernández-Rodríguez A, et al. Kidney transplantation in systemic lupus erythematosus: outcomes and prognosis. Med Clin (Barc). 2019;153(12):460–463. doi:10.1016/j.medcli.2018.09.016. 30502305

[29] Pan L, Lu M-P, Wang J-H, et al. Immunological pathogenesis and treatment of systemic lupus erythematosus. World J Pediatr. 2020;16(1):19–30. doi:10.1007/s12519-019-00229-3. 30796732 PMC7040062

[30] Ramirez-Sandoval JC, Chavez-Chavez H, Wagner M, et al. Long-term survival of kidney grafts in lupus nephritis: a Mexican cohort. Lupus. 2018;27(8):1303–1311. doi:10.1177/0961203318770527. 29697013

[31] Segura BT, Bernstein BS, McDonnell T, et al. Damage accrual and mortality over long-term follow-up in 300 patients with systemic lupus erythematosus in a multi-ethnic British cohort. Rheumatology (Oxford). 2020;59(3):524–533. doi:10.1093/rheumatology/kez292. 31377781 PMC8414923

[32] Sekercioglu N, Fu R. Operations research to solve kidney allocation problems: a systematic review. Healthcare (Basel). 2023;11(5):768. doi:10.3390/healthcare11050768. 36900773 PMC10000664

[33] Sharif A. Deceased donor characteristics and kidney transplant outcomes. Transpl Int. 2022;35:10482. doi:10.3389/ti.2022.10482. 36090778 PMC9452640

[34] Sterne JA, Hernán MA, Reeves BC, et al. ROBINS-I: a tool for assessing risk of bias in non-randomised studies of interventions. BMJ. 2016;355:i4919. doi:10.1136/bmj.i4919. 27733354 PMC5062054

[35] Tektonidou MG, Dasgupta A, Ward MM. Risk of end-stage renal disease in patients with lupus nephritis, 1971-2015: a systematic review and Bayesian meta-analysis. Arthritis Rheumatol. 2016;68(6):1432–1441. doi:10.1002/art.39594. 26815601 PMC5071782

[36] Wells G, Shea B, O’Connell D, et al. The Newcastle-Ottawa Scale (NOS) for assessing the quality of nonrandomized studies in meta-analyses. 2013. http://www.ohri.ca/programs/clinical_epidemiology/oxford.asp.

[37] Wu DA, Watson CJ, Bradley JA, et al. Global trends and challenges in deceased donor kidney allocation. Kidney Int. 2017;91(6):1287–1299. doi:10.1016/j.kint.2016.09.054. 28320531

[38] Yu TM, Chen YH, Lan JL, et al. Renal outcome and evolution of disease activity in Chinese lupus patients after renal transplantation. Lupus. 2008;17(7):687–694. doi:10.1177/0961203308089439. 18625644

[39] Zhang H, Chen J, Zhang Y, et al. Efficacy and safety of belimumab therapy in lupus nephritis: a systematic review and meta-analysis. Ren Fail. n.d.;45(1):2207671. doi:10.1080/0886022X.2023.2207671. 37194710 PMC10193908

[40] Zhang L, Lee G, Liu X, et al. Long-term outcomes of end-stage kidney disease for patients with lupus nephritis. Kidney Int. 2016;89(6):1337–1345. doi:10.1016/j.kint.2016.02.014. 27165824

[41] Zhao H, Alam A, Soo AP, et al. Ischemia-Reperfusion injury reduces long term renal graft survival: mechanism and Beyond. EBioMedicine. 2018;28:31–42. doi:10.1016/j.ebiom.2018.01.025. 29398595 PMC5835570

