# Supplementary material for: Graft survival and mortality outcomes after kidney transplant in patients with lupus nephritis: a systematic review and meta-analysis
Source: Ren Fail. 2024 Jan 4;46(1):2296000. doi: 10.1080/0886022X.2023.2296000 (PMC10773647; doi:10.1080/0886022X.2023.2296000)

**Supplementary table 1. Search strategy used**

| A) PubMed |
|---|
| #1 (lupus nephritis OR SLE OR autoimmune OR Lupus Erythematosus) AND (renal transplant OR kidney transplant)<br>#2 (renal transplant OR renal transplant)<br>#3 (clinical outcomes OR patient survival OR graft survival OR overall survival)<br>#4 (#1 AND #2 AND #3)<br>#5 (Addresses[ptyp] OR Autobiography[ptyp] OR Bibliography[ptyp] OR Biography[ptyp] OR pubmed books[filter] OR Case Reports[ptyp] OR Congresses[ptyp] OR Consensus Development Conference[ptyp] OR Directory[ptyp] OR Duplicate Publication[ptyp] OR Editorial[ptyp] OR Systematic reviews OR Meta analysis OR Festschrift[ptyp] OR Guideline[ptyp] OR In Vitro[ptyp] OR Interview[ptyp] OR Lectures [ptyp] OR Legal Cases[ptyp] OR News[ptyp] OR Newspaper Article[ptyp] OR Personal Narratives [ptyp] OR Portraits[ptyp] OR Retracted Publication[ ptyp] OR Twin Study[ptyp] OR Video-Audio Media[ptyp])<br><br>#6 (#4 NOT #5) |

| B) Embase |
|---|
| (lupus nephritis or SLE or autoimmune or Lupus Erythematosus or lupus*) and (renal transplant or kidney transplant) AND 'lupus':ti,ab,kw or “autoimmune’; ti,ab,kw or “survival”: ti,ab,kw or “graft rejection”:ti,ab,kw |

| C) Scopus |
|---|
| #1 (lupus nephritis) or (autoimmune) or (SLE) OR TITLE-ABS-KEY (lupus nephritis)<br>#2 (renal transplant) or (kidney transplant) or (transplant) OR TITLE-ABS-KEY (renal transplant)<br>#3 (survival) or (graft survival) or (graft rejection) OR TITLE-ABS-KEY (outcomes) #1 and #2 and #3 |

**Supplementary table 2. References of the studies excluded at the time of full text review**

1. López-Morales JM, Quintanilla-González L, Ramírez-Sandoval JC, Hinojosa-Azaola A. Early outcomes in kidney transplant recipients with systemic lupus erythematosus. Rheumatol Int. 2019 Mar;39(3):479-487. doi: 10.1007/s00296-018-4234-7.
2. Stone JH, Amend WJ, Criswell LA. Outcome of renal transplantation in systemic lupus erythematosus. Semin Arthritis Rheum. 1997 Aug;27(1):17-26. doi: 10.1016/s00490172(97)80033-9. PMID: 9287386.
3. Lochhead KM, Pirsch JD, D'Alessandro AM, Knechtle SJ, Kalayoglu M, Sollinger HW, Belzer FO. Risk factors for renal allograft loss in patients with systemic lupus erythematosus. Kidney Int. 1996 Feb;49(2):512-7.
4. Azevedo LS, Romão JE Jr, Malheiros D, Saldanha LB, Ianhez LE, Sabbaga E. Renal transplantation in systemic lupus erythematosus. A case control study of 45 patients. Nephrol Dial Transplant. 1998 Nov;13(11):2894-8.
5. Grimbert P, Frappier J, Bedrossian J, Legendre C, Antoine C, Hiesse C, Bitker MO, Sraer JD, Lang P. Long-term outcome of kidney transplantation in patients with systemic lupus erythematosus: a multicenter study. Groupe Cooperatif de Transplantation d'île de France. Transplantation. 1998 Oct 27;66(8):1000-3.
6. Stone JH, Amend WJ, Criswell LA. Outcome of renal transplantation in ninety-seven cyclosporine-era patients with systemic lupus erythematosus and matched controls. Arthritis Rheum. 1998 Aug;41(8):1438-45.
7. Mai K, Singer P, Fahmy AE, Teperman LW, Molmenti EP, Grodstein EI, Castellanos L, Sethna CB. Kidney transplant outcomes in children and adolescents with systemic lupus erythematosus. Pediatr Transplant. 2022 Feb;26(1):e14178. doi: 10.1111/petr.14178.
8. Ward MM. Outcomes of renal transplantation among patients with end-stage renal disease caused by lupus nephritis. Kidney Int. 2000 May;57(5):2136-43.
9. Ntatsaki E, Velo-Garcia A, Vassiliou VS, Salama AD, Isenberg DA. Impact of pre-transplant time on dialysis on survival in patients with lupus nephritis. Clin Rheumatol. 2018 Sep;37(9):2399-2404.
10. Albuquerque BC, Salles VB, Tajra RDP, Rodrigues CEM. Outcome and Prognosis of Patients With Lupus Nephritis Submitted to Renal Transplantation. Sci Rep. 2019 Aug 12;9(1):11611. doi: 10.1038/s41598-019-48070-y.
11. Wagner CS, et al. Outcomes in renal transplant recipients with lupus nephritis: experience at a single center. *Ren Fail.* 2014;**36**:912–915.
12. Park DJ, Joo YB, Bang SY, Lee J, Lee HS, Bae SC. Predictive Factors for Renal Response in Lupus Nnorephritis: A Single-center Prospective Cohort Study. J Rheum Dis. 2022 Oct 1;29(4):223-231
13. Fuentes L, et al. Survival of lupus nephritis patients after renal transplantation In Malaga. *Transplant Proc.* 2012;**44**:2067–2068. doi: 10.1016/j.transproceed.2012.07.063.
14. Azevedo LS, et al. Renal transplantation in systemic lupus erythematosus. A case control study of 45 patients. *Nephrol Dial Transplant.* 1998;**13**:2894–2898.

15. Chan EY, Yap DY, Wong WT, Wong WH, Wong SW, Lin KY, Hui FY, Yee-Ming J, Lam SS, Wong JK, Lai FF, Ho TW, Tong PC, Lai WM, Chan TM, Ma AL. Long-Term Outcomes of Children and Adolescents With Biopsy-Proven Childhood-Onset Lupus Nephritis. Kidney Int Rep. 2022 Oct 21;8(1):141-150.

16. Demir S, Gülhan B, Özen S, Çeleğen K, Batu ED, Taş N, Orhan D, Bilginer Y, Düzova A, Ozaltin F, Topaloğlu R. Long-term renal survival of paediatric patients with lupus nephritis. Nephrol Dial Transplant. 2022 May 25;37(6):1069-1077.

17. Goss JA, Cole BR, Jendrisak MD, McCullough CS, So SK, Windus DW, Hanto DW. Renal transplantation for systemic lupus erythematosus and recurrent lupus nephritis. A singlecenter experience and a review of the literature. Transplantation. 1991 Nov;52(5):805-10.

18. Cairoli E, Sanchez-Marcos C, Espinosa G, Glucksmann C, Ercilla G, Oppenheimer F, Cervera R. Renal transplantation in systemic lupus erythematosus: outcome and prognostic factors in 50 cases from a single centre. Biomed Res Int. 2014;2014:746192. doi: 10.1155/2014/746192.

19. Pattanaik D, Green J, Talwar M, Molnar M. Relapse and Outcome of Lupus Nephritis After Renal Transplantation in the Modern Immunosuppressive Era. Cureus. 2022 Jan 1;14(1):e20863. doi: 10.7759/cureus.20863

20. Hocaoğlu M, Valenzuela-Almada MO, Dabit JY, Osei-Onomah SA, Chevet B, Giblon RE, Zand L, Fervenza FC, Helmick CG, Crowson CS, Duarte-García A. Incidence, Prevalence, and Mortality of Lupus Nephritis: A Population-Based Study Over Four Decades Using the Lupus Midwest Network. Arthritis Rheumatol. 2023 Apr;75(4):567-573.

21. Contreras G, Mattiazzi A, Guerra G, Ortega LM, Tozman EC, Li H, Tamariz L, Carvalho C, Kupin W, Ladino M, LeClercq B, Jaraba I, Carvalho D, Carles E, Roth D. Recurrence of lupus nephritis after kidney transplantation. J Am Soc Nephrol. 2010 Jul;21(7):1200-7. doi: 10.1681/ASN.2009101093.

22. Goral S, Ynares C, Shappell SB, Snyder S, Feurer ID, Kazancioglu R, Fogo AB, Helderman JH. Recurrent lupus nephritis in renal transplant recipients revisited: it is not rare. Transplantation. 2003 Mar 15;75(5):651-6. doi: 10.1097/01.TP.0000053750.59630.83.

23. Nyberg G, Blohmé I, Persson H, Olausson M, Svalander C. Recurrence of SLE in transplanted kidneys: a follow-up transplant biopsy study. Nephrol Dial Transplant. 1992;7(11):1116-23. PMID: 1336139.

24. Burgos PI, Perkins EL, Pons-Estel GJ, Kendrick SA, Liu JM, Kendrick WT, Cook WJ, Julian BA, Alarcón GS, Kew CE 2nd. Risk factors and impact of recurrent lupus nephritis in patients with systemic lupus erythematosus undergoing renal transplantation: data from a single US institution. Arthritis Rheum. 2009 Sep;60(9):2757-66.

25. Mojcik CF, Klippel JH. End-stage renal disease and systemic lupus erythematosus. Am J Med. 1996 Jul;101(1):100-7. doi: 10.1016/s0002-9343(96)00074-5.

26. Jorge A, Wallace ZS, Lu N, Zhang Y, Choi HK. Renal Transplantation and Survival Among Patients With Lupus Nephritis: A Cohort Study. Ann Intern Med. 2019 Feb 19;170(4):240247. doi: 10.7326/M18-1570. Epub 2019 Jan 22.

27. Ponticelli C, Moroni G. Renal transplantation in lupus nephritis. Lupus. 2005;14(1):95-8. doi: 10.1191/0961203305lu2067oa.

28. Gołębiewska J, Dębska-Ślizień A, Bułło-Piontecka B, Rutkowski B. Outcomes in Renal Transplant Recipients With Lupus Nephritis-A Single-Center Experience and Review of the Literature. Transplant Proc. 2016 Jun;48(5):1489-93.

29. Clark WF, Jevnikar AM. Renal transplantation for end-stage renal disease caused by systemic lupus erythematosus nephritis. Semin Nephrol. 1999 Jan;19(1):77-85

30. Oniscu GC, Brown H, Forsythe JL. Impact of cadaveric renal transplantation on survival in patients listed for transplantation. J Am Soc Nephrol. 2005 Jun;16(6):1859-65.

31. Naveed A, Nilubol C, Melancon JK, Girlanda R, Johnson L, Javaid B. Preemptive kidney transplantation in systemic lupus erythematosus. Transplant Proc. 2011 Dec;43(10):3713-4.

**Supplementary Figure 1. Risk of bias assessment of the included studies using the ROBINS-I tool**

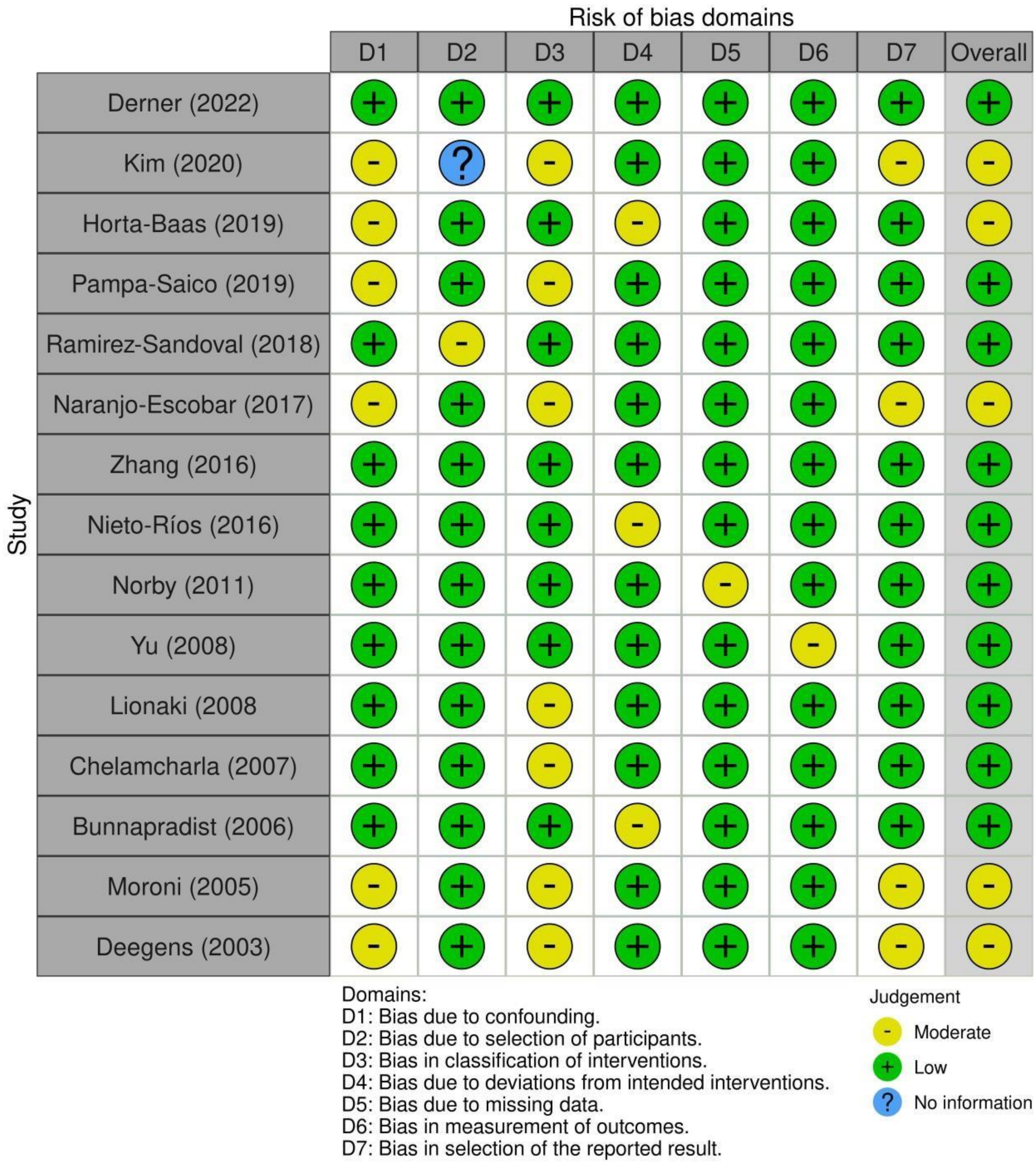
Risk of bias domains
D1 D2 D3 D4 D5 D6 D7 Overall
Study
Derner (2022) + + + + + + + +
Kim (2020) - ? - + + + - -
Horta-Baas (2019) - + + - + + + -
Pampa-Saico (2019) - + - + + + + +
Ramirez-Sandoval (2018) + - + + + + + +
Naranjo-Escobar (2017) - + - + + + - -
Zhang (2016) + + + + + + + +
Nieto-Ríos (2016) + + + - + + + +
Norby (2011) + + + + - + + +
Yu (2008) + + + + + - + +
Lionaki (2008 + + - + + + + +
Chelamcharla (2007) + + - + + + + +
Bunnapradist (2006) + + + - + + + +
Moroni (2005) - + - + + + - -
Deegens (2003) - + - + + + - -
Domains:
D1: Bias due to confounding.
D2: Bias due to selection of participants.
D3: Bias in classification of interventions.
D4: Bias due to deviations from intended interventions.
D5: Bias due to missing data.
D6: Bias in measurement of outcomes.
D7: Bias in selection of the reported result.
Judgement
- Moderate
+ Low
? No information

**Supplementary Figure 2**

**. Funnel plot for publication bias comparing patient survival among those with lupus nephritis undergoing renal transplant and those with end-stage renal disease due to other causes**

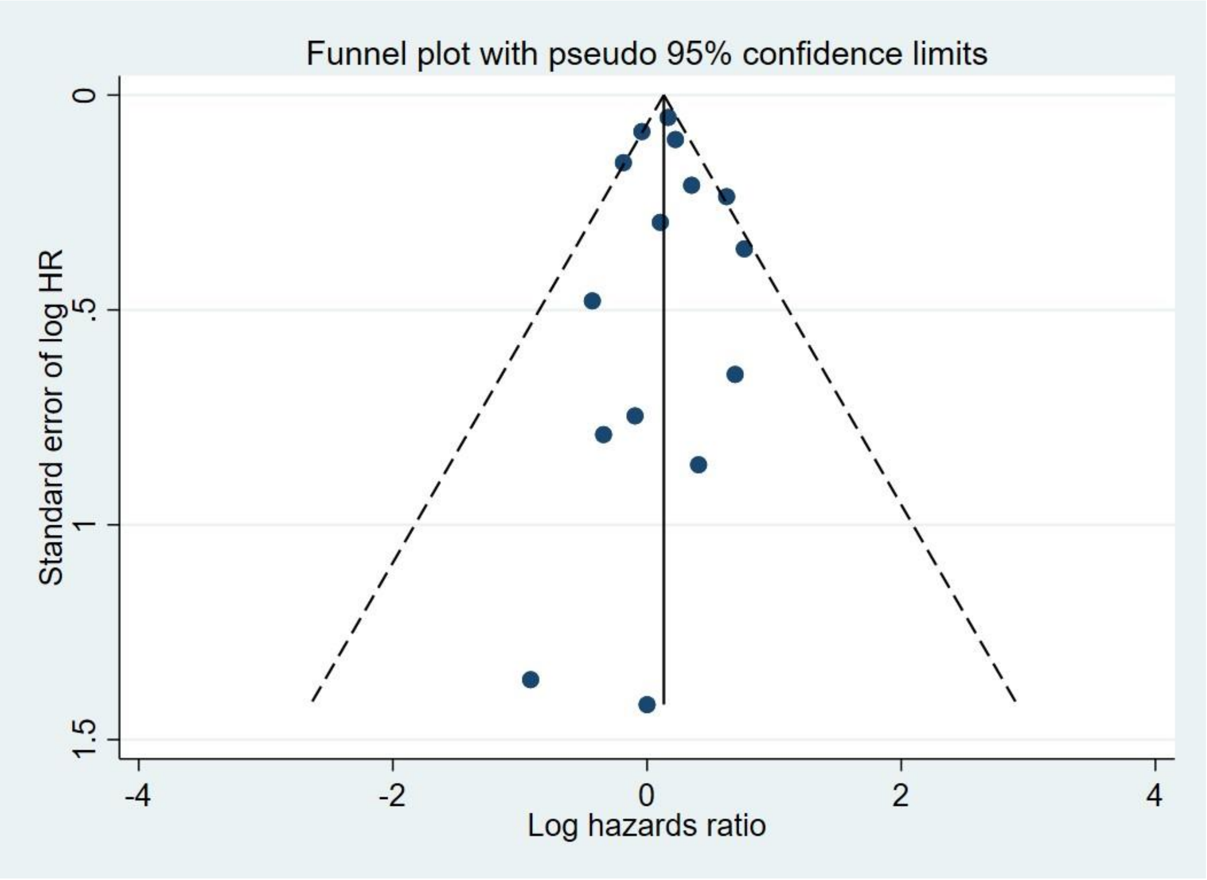

**Supplementary Figure 3**

**. Forest plot for subgroup analysis based on sample size (≤200 and >200) comparing patient survival among those with lupus nephritis undergoing renal transplant and those with end-stage renal disease due to other causes**

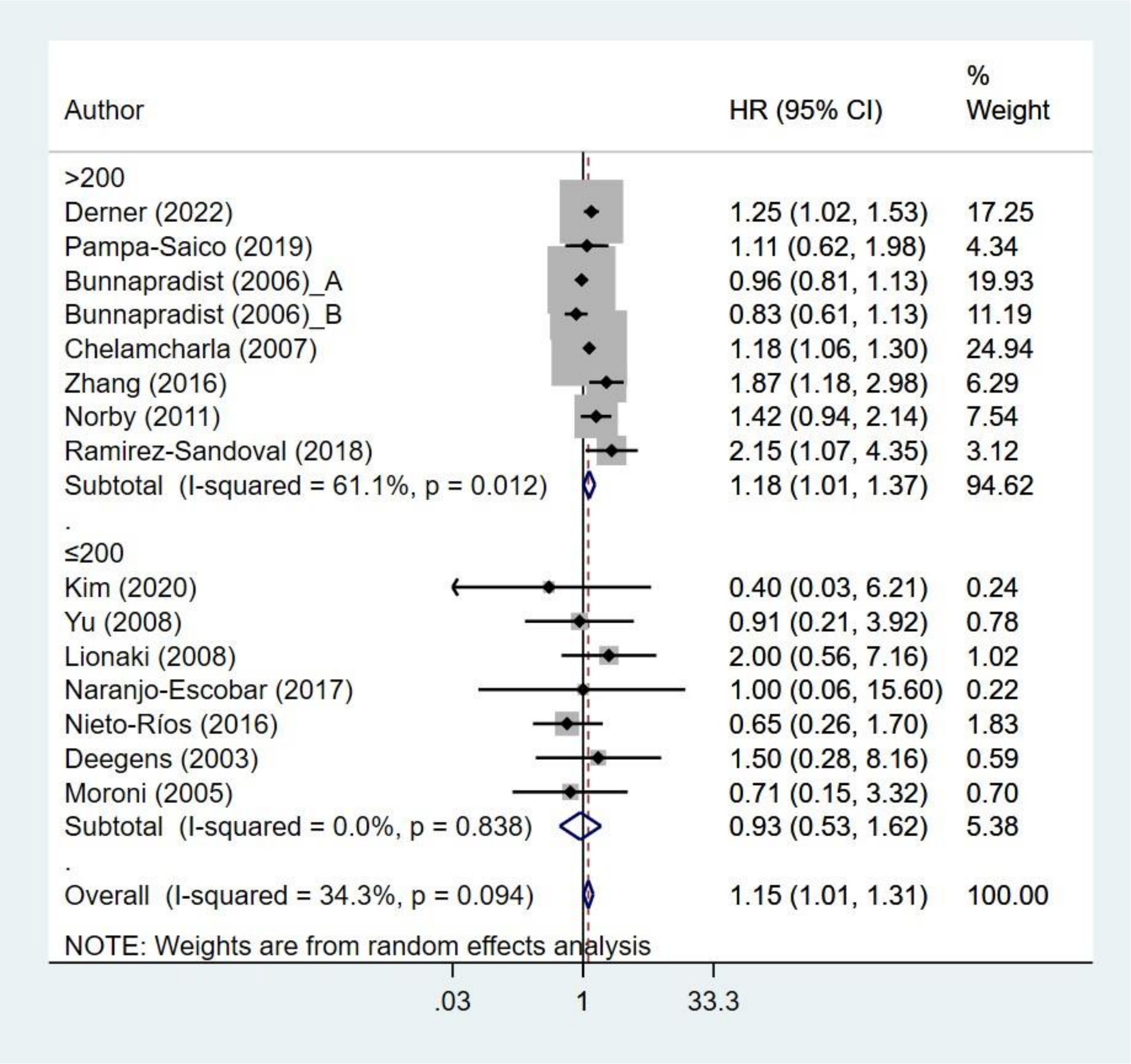

**Supplementary Figure 4**

**. Forest plot for subgroup analysis based on study design (retrospective cohort, RC and case-control, CC) comparing patient survival among those with lupus nephritis undergoing renal transplant and those with end-stage renal disease due to other causes**

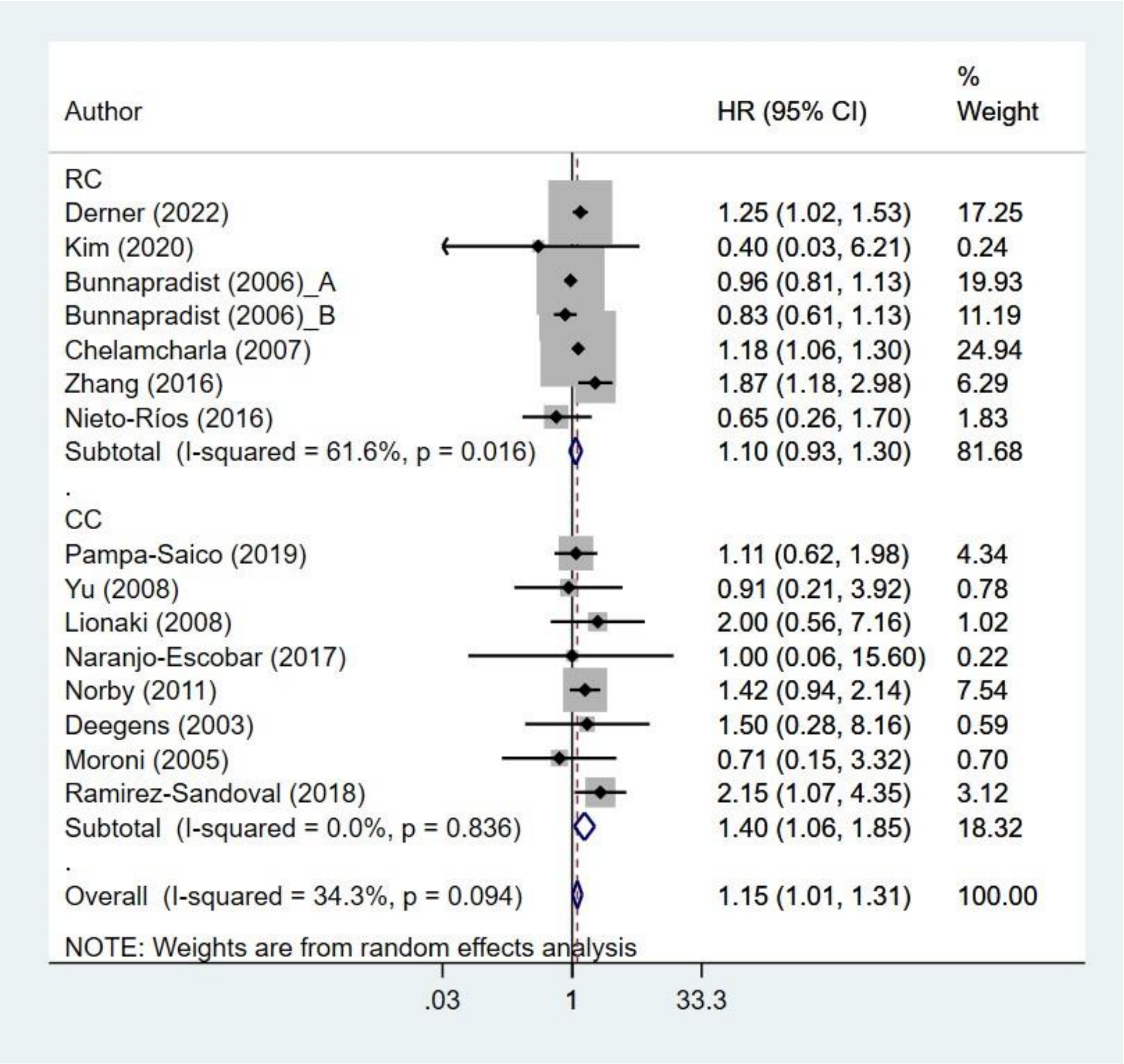

**Supplementary Figure 5**

**. Funnel plot for publication bias comparing graft survival among those with lupus nephritis undergoing renal transplant and those with end-stage renal disease due to other causes**

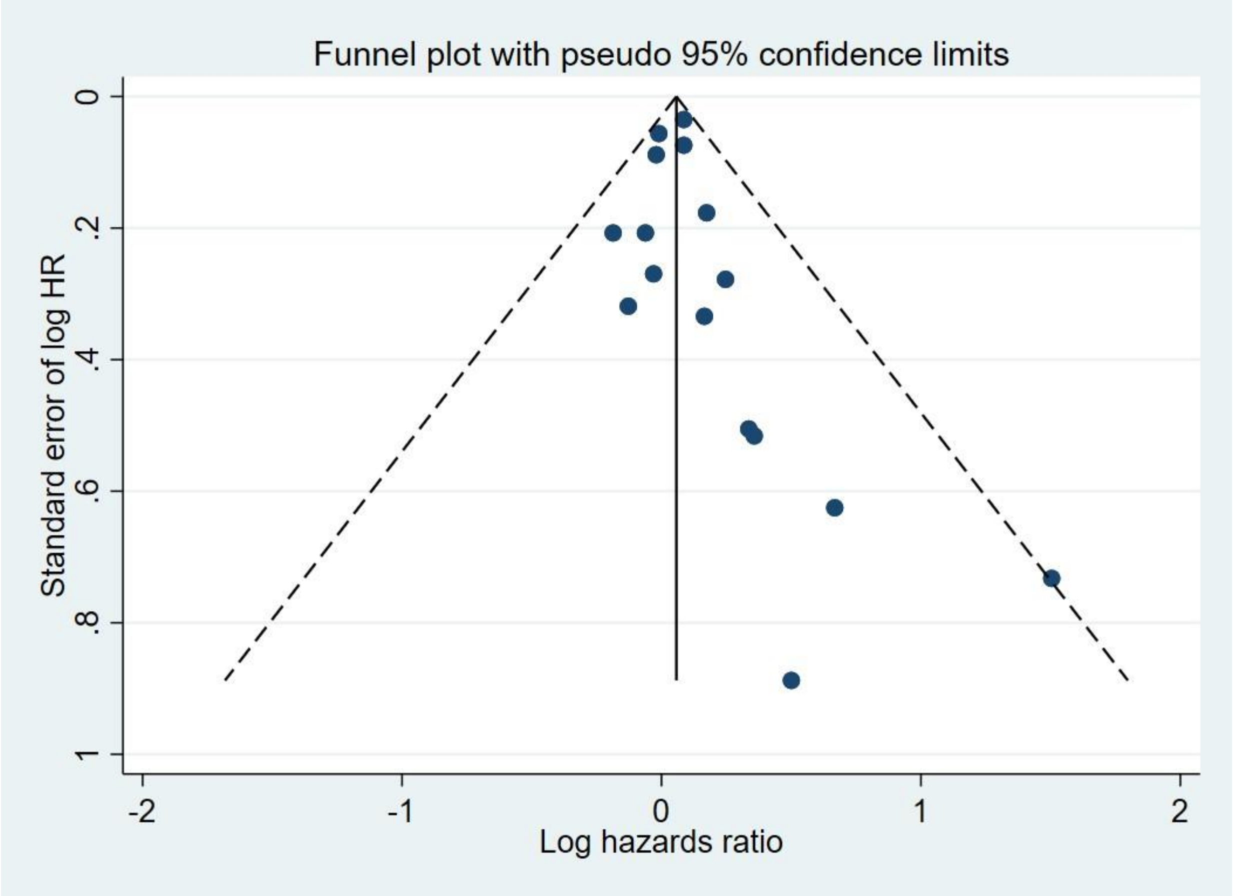

**Supplementary Figure 6**

**. Forest plot for subgroup analysis based on sample size (≤200 and >200) comparing graft survival among those with lupus nephritis undergoing renal transplant and those with end-stage renal disease due to other causes**

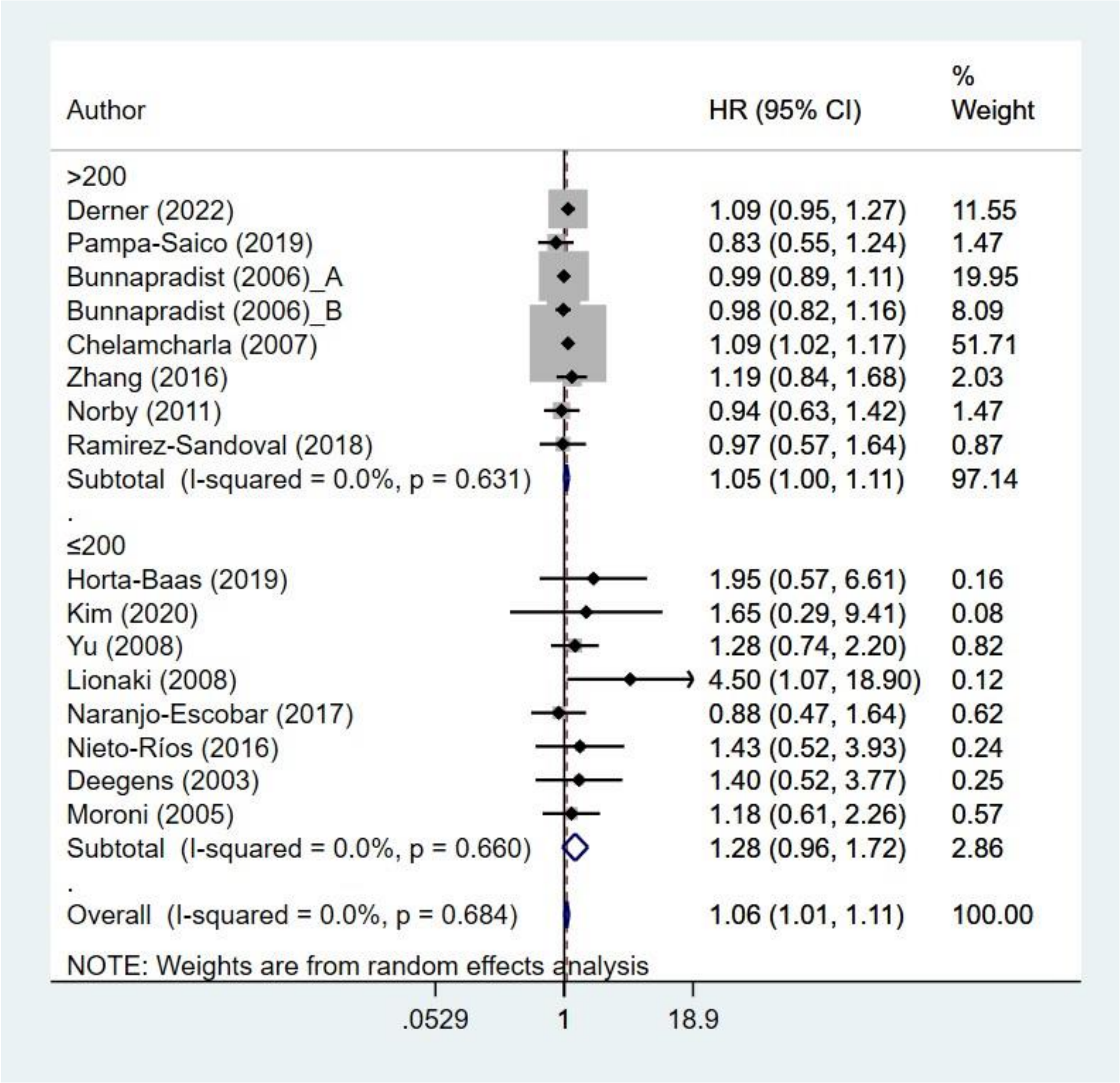

**Supplementary Figure 7**

. **Forest plot for subgroup analysis based on study design (retrospective cohort, RC and case-control, CC) comparing graft survival among those with lupus nephritis undergoing renal transplant and those with end-stage renal disease due to other causes**

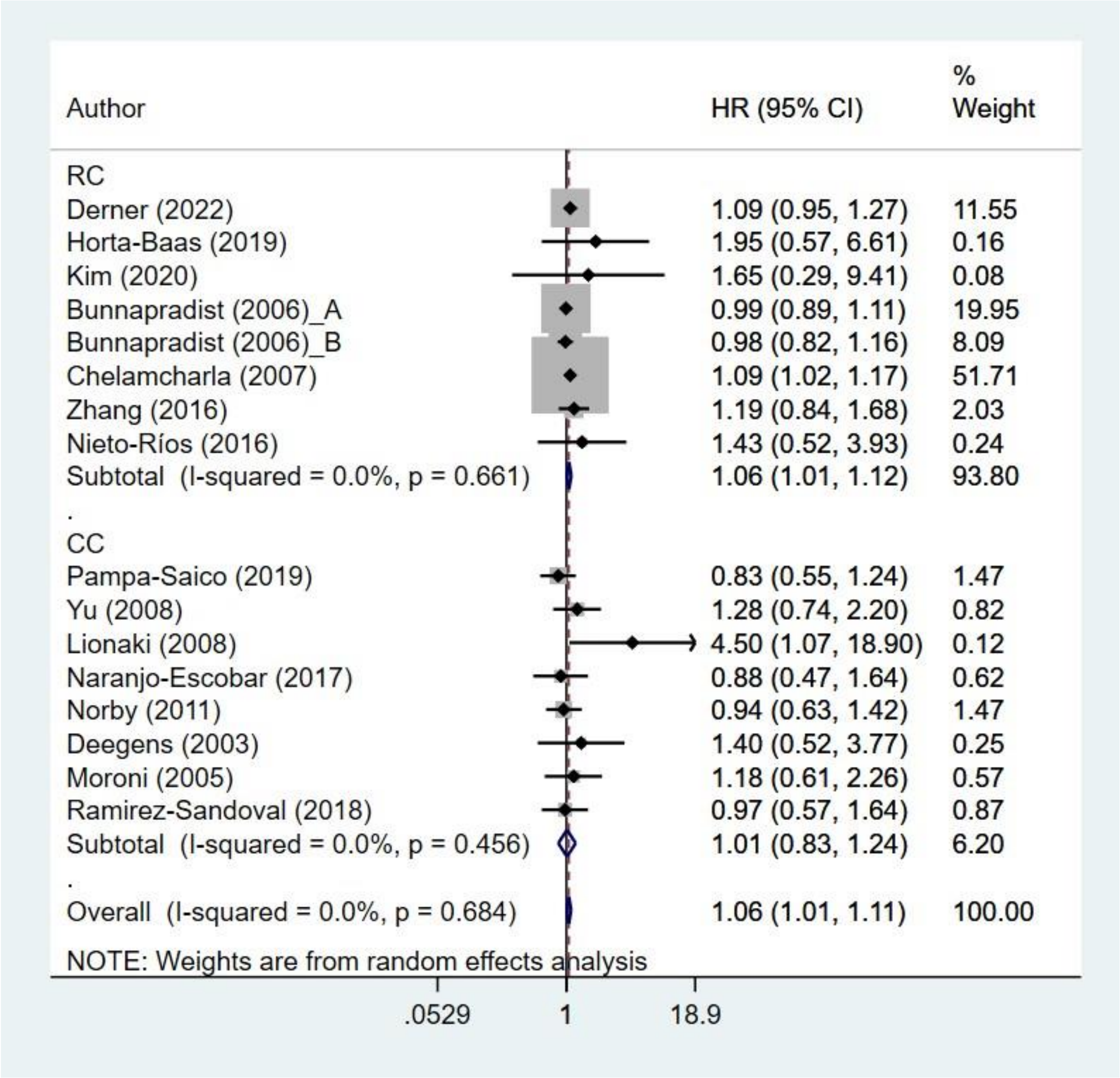

Supplement: Supplemental Material [file IRNF_A_2296000_SM5063.pdf]
